# Patterns of Transcript Abundance of Eukaryotic Biogeochemically-Relevant Genes in the Amazon River Plume

**DOI:** 10.1371/journal.pone.0160929

**Published:** 2016-09-06

**Authors:** Brian L. Zielinski, Andrew E. Allen, Edward J. Carpenter, Victoria J. Coles, Byron C. Crump, Mary Doherty, Rachel A. Foster, Joaquim I. Goes, Helga R. Gomes, Raleigh R. Hood, John P. McCrow, Joseph P. Montoya, Ahmed Moustafa, Brandon M. Satinsky, Shalabh Sharma, Christa B. Smith, Patricia L. Yager, John H. Paul

**Affiliations:** 1 University of South Florida College of Marine Science, St. Petersburg, FL, United States of America; 2 Department of Microbial and Environmental Genomics, J. Craig Venter Institute, San Diego, CA, United States of America; 3 Romberg Tiburon Center, San Francisco State University, Tiburon, California, United States of America; 4 Horn Point Laboratory, University of Maryland Center for Environmental Science, Cambridge, MD, United States of America; 5 College of Earth, Ocean, and Atmospheric Sciences, Oregon State University, Corvallis, Oregon, United States of America; 6 Rhodes College, Memphis, TN, United States of America; 7 Ocean Sciences, University of California, Santa Cruz, CA, United States of America; 8 Lamont Doherty Earth Observatory, Columbia University, Palisades, NY, United States of America; 9 School of Biology, Georgia Institute of Technology, Atlanta, GA, United States of America; 10 Department of Biology and Biotechnology Graduate Program, American University in Cairo, New Cairo, Egypt; 11 Department of Civil and Environmental Engineering, Massachusetts Institute of Technology, Cambridge, MA, United States of America; 12 Department of Marine Sciences, University of Georgia, Athens, GA, United States of America; 13 Department of Ecology, Environment, and Plant Sciences, Stockholm University, Stockholm, Sweden; Universidad Miguel Hernandez de Elche, SPAIN

## Abstract

The Amazon River has the largest discharge of all rivers on Earth, and its complex plume system fuels a wide array of biogeochemical processes, across a large area of the western tropical North Atlantic. The plume thus stimulates microbial processes affecting carbon sequestration and nutrient cycles at a global scale. Chromosomal gene expression patterns of the 2.0 to 156 μm size-fraction eukaryotic microbial community were investigated in the Amazon River Plume, generating a robust dataset (more than 100 million mRNA sequences) that depicts the metabolic capabilities and interactions among the eukaryotic microbes. Combining classical oceanographic field measurements with metatranscriptomics yielded characterization of the hydrographic conditions simultaneous with a quantification of transcriptional activity and identity of the community. We highlight the patterns of eukaryotic gene expression for 31 biogeochemically significant gene targets hypothesized to be valuable within forecasting models. An advantage to this targeted approach is that the database of reference sequences used to identify the target genes was selectively constructed and highly curated optimizing taxonomic coverage, throughput, and the accuracy of annotations. A coastal diatom bloom highly expressed nitrate transporters and carbonic anhydrase presumably to support high growth rates and enhance uptake of low levels of dissolved nitrate and CO_2_. Diatom-diazotroph association (DDA: diatoms with nitrogen fixing symbionts) blooms were common when surface salinity was mesohaline and dissolved nitrate concentrations were below detection, and hence did not show evidence of nitrate utilization, suggesting they relied on ammonium transporters to aquire recently fixed nitrogen. These DDA blooms in the outer plume had rapid turnover of the photosystem D1 protein presumably caused by photodegradation under increased light penetration in clearer waters, and increased expression of silicon transporters as silicon became limiting. Expression of these genes, including carbonic anhydrase and transporters for nitrate and phosphate, were found to reflect the physiological status and biogeochemistry of river plume environments. These relatively stable patterns of eukaryotic transcript abundance occurred over modest spatiotemporal scales, with similarity observed in sample duplicates collected up to 2.45 km in space and 120 minutes in time. These results confirm the use of metatranscriptomics as a valuable tool to understand and predict microbial community function.

## Introduction

The Amazon River discharges an average of 1.55 × 10^5^ ± 0.13 m^3^ s^-1^ at Obidos, Brazil, ultimately resulting in a thin, fresh water layer at the surface called the Amazon River plume (ARP), which also varies seasonally and covers up to 1.3 × 10^6^ km^2^ of the western tropical North Atlantic Ocean [1–4]. The ARP harbors many distinct microbial communities along the salinity gradient [5]. In lower salinity waters, where dissolved nutrients such as silica, iron, nitrate and phosphate are abundant, coastal diatom communities flourish once light can penetrate the initially turbid plume [4, 6]. Once ammonium and nitrate are depleted in the mesohaline portions of the plume, diatom-diazotroph association (DDA) blooms utilize the remaining silica while endosymbiotic cyanobacteria fix nitrogen and transfer it to the diatoms [4, 7]. There are at least 4 genera of diatoms (*Hemiaulus*, *Rhizosolenia*, *Chaetocerous*, *Guinardia*) which form partnerships, or symbioses, with nitrogen fixing heterocystous cyanobacteria (*Richelia intracellularis* and *Calothrix rhizosoleniae)* and collectively these are referred to as DDAs [8]. These DDA blooms exhibit high rates of nitrogen and carbon fixation worldwide [4, 9]. DDA blooms in the ARP sequester 1.7 Tmol of carbon annually [4], and similar distributions of DDAs have been reported in the Niger and Congo river plumes as well as the South China Sea [10, 11]. When silica and phosphate are no longer available, nitrogen-fixing *Trichodesmium* dominate, which have been shown to regulate their buoyancy with gas vesicles to acquire phosphorus at depth [12].

Metatranscriptomics, the collection and analysis of mRNA from a community of organisms, allows us to study fluctuations in the molecular response of natural communities to changing environmental conditions. The first report to describe an environmental metatranscriptome was that of Poretsky et al. who built primarily prokaryotic mRNA libraries derived from Sapelo Island Microbial Observatory (SIMO, a tidal creek in a salt marsh) and the Mono Lake Microbial Observatory (MLMO, a hypersaline soda lake) [13]. Metatranscriptomics can elucidate how communities respond to environmental changes, including, for example, temperature effects on eukaryotic phytoplankton metabolism [14], oil spill impacts on deep bacterioplankton [15], and differences in free-living or particle-associated habitats [16]. Although much has been published about river plume communities [6, 17–20], including some findings about individual gene expression [21], metatranscriptomic analysis of microbial eukaryotes has not yet been performed to examine the difference in gene expression as a measure of metabolic activity along a river plume salinity gradient.

Reported here are findings from part of two large, multi-investigator research programs: the River Ocean Continuum of the Amazon (*ROCA*) and Amazon iNfluence of the Atlantic: CarbOn export from Nitrogen fixation by DiAtom Symbioses (*ANACONDAS*). We have used metatranscriptomics to show how the expression of 31 genes relates to the nitrogen, silica, phosphate, and carbon gradients in the ARP. Focusing a large dataset on minimal genes is crucial to make models where the appropriate caluclations can be performed across the entirety of the tropical Atlantic. In particular, these 31 genes enable inferences to be drawn on the physiologic status of communities such as the DDAs. We also demonstrate that replicates taken up to two hours and 2.4 km apart still show similar patterns of gene expression across the 31 genes analyzed. Modeling efforts are currently using these data to expand predictive capabilities of their ARP ecological models [22].

## Materials and Methods

A detailed discussion of sample collection, DNA and mRNA processing, sequencing, and metadata collection can be found in a sequence-release announcement [23]. DNA samples were taken at the same stations, but with different filters. DNA methods are fully described in the sequence-release announcement [23]. These methods and the location of the metadata used are briefly summarized below.

### Sample Collection

All samples reported here were collected during the May-June 2010 *ANACONDAS* expedition onboard the U.S. RV *Knorr* (KN-197-8; http://www.bco-dmo.org/project/2097). At each of the six stations selected for metatranscript analysis, near-surface water (upper 3 m) was collected at about the same time of day (just after local sunrise) by gentle impeller pumping (modified Rule 1800 submersible pump) through 10 m of Tygon tubing (3 cm diameter) to the ship’s deck where the water was pre-filtered through a 156 μm mesh into 20 L carboys. The water was immediately taken to the shipboard laboratory, and gently filtered (using a Masterflex peristaltic pump) through a 2.0 μm pore-size, 142 mm diameter polycarbonate (PCTE) membrane filter (Sterlitech Corporation, Kent, CWA). After < 30 minutes of filtration, membranes were submerged in RNAlater (Applied Biosystems, Austin, TX) in sterile 50 ml conical tubes, incubated at room temperature for at least 4 hours, stored onboard at -80°C, shipped in liquid nitrogen, and archived at -80°C until RNA extraction. All filtration and fixation was completed within 30 min of water collection, and the volume of filtrate passed through each membrane was recorded. A second (duplicate) sample was collected similarly for each station in the same general area (within 2.5 km) within two hours of the first sample.

### RNA Processing for Eukaryotic Metatranscriptomes

Prior to RNA extraction, filters were thawed, removed from the preservative solution, placed in Whirl-Pak bags (Nasco, Fort Artkinson, WI), and flash-frozen in liquid nitrogen. A lysis tube was prepared for each sample consisting of a sterile 50 ml conical tube containing 10 ml of RLT Lysis Solution and 1.5 ml of 100 μm zirconium beads (OPS Diagnostics, Lebanon, NJ, USA). The brittle filters inside the bags were broken into small pieces using a rubber mallet and transferred to the lysis tubes. Tubes were vortexed for 10 min to lyse cells, and RNA was purified from cell lysate using an RNeasy Kit (Qiagen, Valencia, CA). Following lysis, poly(A)-tailed mRNA was isolated from total RNA using an Oligotex mRNA kit (Qiagen, Valencia, CA), and mRNA was linearly amplified with one round of the MessageAmp II aRNA Amplification Kit (Applied Biosystems, Austin, TX). mRNA was converted into cDNA using the Superscript III First Strand synthesis system (Invitrogen, Carlsbad, CA) with random primers, followed by the NEBnext mRNA second strand synthesis module (New England Biolabs, Ipswich, MA), both according to manufacturer protocols. The 3’ adenine overhang was removed with T4 polymerase and the cDNA was purified using the DNA Clean and Concentrator -25 Kit (Zymo, Irvine, CA) with five volumes of DNA binding buffer. DNA was resuspended in 100 μL of TE buffer, and stored at -80°C until sample preparation for sequencing.

### Sequencing

cDNA was sheared ultrasonically to ~200–250 bp fragments and TruSeq libraries (Illumina Inc., San Diego, CA) were constructed for paired-end (150 x 150) sequencing using the Illumina Genome Analyzer IIx, HiSeq2000, MiSeq, or HiSeq2500 platforms (Illumina Inc., San Diego, CA).

### Bioinformatics

Paired-end reads were joined using the She-ra program [24] with a quality metric score of 0.5. Paired reads were trimmed using Seqtrim 20 [25]. Poly(a)-RNA capture methods were not 100% successful, as some rRNA would remain in the sample, so the minimal (0.14–0.33%) rRNA were removed in silico after sequencing. Remaining rRNA reads in the metatranscriptomes were removed via a Blastn against a database containing rRNA sequences from Genbank. Reads with a bit score >50 to one of the sequences in the database were removed. Reads representing genes or transcripts of 31 selected proteins (database described below) were identified using a Blastx search with a bit score cutoff of 40 against a custom database consisting of multiple reference sequences from diverse taxa for each gene, along with their paralogs to eliminate false-positives. Sequences that hit the targeted gene database were subsequently queried against RefSeq protein database using Blastx to confirm the functional assignment of the reads and to obtain taxanomic designations.

The custom database used contained thirty-one well characterized genes representative of key biological functionality such as carbon autotrophy and heterotrophy and nitrogen, phosphorus, sulfur and silicon cycling. Ten to twenty amino acid sequences covering a broad range of taxonomy were used as reference sequences for each protein, and sequences representative of paralogs for the selected genes were also included in the database to eliminate false-positives. As often as possible, genes were collected directly from physiological papers where the specific sequences were originally identified and sequenced. This gene-specific reference database was tested on a subset of Amazon reads using a bit score >40, and a re-analysis of the positive reads against the RefSeq protein database was used to adjust the composition of the database.

Metagenomic sample collection and processing were performed by collaborators, and detailed methodology has been previously published [23]. True replicates were utilized at each station to collect the metagenomics samples. Metagenomic sequences were searched for 18S rDNA candidates via matching for a 18S reference covariance model using Infernal [26]. A Blastn [27] search was performed against SILVA [28] (release 115) to identify study-specific taxa to be included in the reference tree, in addition to a predefined set of core sequences representing the major eukaryotic lineages. The search was executed on a TimeLogicDeCypher system (Active Motif Inc., Carlsbad, CA) with *e*-value threshold ≤ 1E-100. Reference sequences were then aligned with MAFFT [29] using the G-INS-i setting for global homology. The generated multiple sequence alignment was visually inspected and manually edited and refined using JalView [30]. A maximum likelihood reference tree was inferred under the general time-reversible model with gamma-distributed rate heterogeneity and an estimated proportion of invariant sites (GTR + Γ + I), implemented in RAxML [31] and the bootstrap support values assessed with the extended majority-rule consensus tree (autoMRE) criterion [32]. The predicted metagenomic 18S rDNA sequences were mapped onto the reference tree using pplacer [33] with the default settings. The counts of the sequences affiliated with the nodes on the reference tree were normalized to the total number of sequences from their corresponding samples. The normalized abundances are visualized as circles mapped on the reference tree such that the diameters of the circles are proportion to the taxonomic abundances. No corrections were applied to account for a difference in 18S copy number per species.

Nitrate transporters (*NRT*) were analyzed by performing hmmsearch [34] for the NCBI CDD nitrate transmembrane transporter models, PLN00028 and NarK (COG2223) for eukarya and bacteria, respectively. Similarly, hmmsearch was performed against a comprehensive reference database compiled from NCBI RefSeq [35] (release 60), microbial eukaryotic genomes from JGI (http://genome.jgi-psf.org/), and the recently released microbial eukaryotic transcriptomic libraries by MMETSP (http://marinemicroeukaryotes.org/). The sequences from the reference databases were used to infer a reference phylogenetic tree for the *NRT*s. Reference sequences were aligned with MAFFT [29] using the E-INS-i setting for multiple conserved domains and long gaps. The multiple sequence alignment was visually inspected and manually refined using JalView [30]. A maximum likelihood reference tree was inferred under the WAG model for amino acid substitutions [36] with gamma-distributed rate heterogeneity and an estimated proportion of invariant sites (WAG + Γ + I), implemented in FastTree [37]. The branch confidence values were estimated using the Shimodaira-Hasegawa (SH) test [38] with 1,000 resampling replicates. The *NRT* environmental ORFs were mapped onto the reference tree using pplacer [33] with the default settings. The numbers of ORFs affiliated with the nodes on the reference tree were normalized to the total number of reads from the corresponding samples. The normalized expression levels are visualized as circles mapped on the reference tree such that the diameters of the circles are proportional to the expression levels.

### Sequence Availability

Sequences are available from the iMicrobe (data.imicrobe.us) database under project number CAM_P_0001194. The sequences are quality controlled fasta files of joined paired-end reads following removal of rRNA sequences (metatranscriptomes only). Sequences are also available from NCBI under accession numbers [SRP039390] (metagenomes) and [SRP039544] (poly(A)-selected metatranscriptomes). The NCBI sequences are fastq files from which rRNA sequences (metatranscriptomes only) have been removed prior to deposition.

### Metadata

Environmental data (temperature, salinity, beam transmittance, dissolved oxygen, pCO_2_, etc.) have been previously published [2, 23], as have nutrient and community structure data corresponding to the stations examined [5, 23, 39, 40]. The abundances of the diazotroph population was determined by epifluorescence microscopy as previously described [41]. ANACONDAS and ROCA project data are also available at the BCO-DMO data repository (http://www.bco-dmo.org/project/2097).

## Results and Discussion

### Hydrographic Conditions and Community Structure

**Fig 1:**
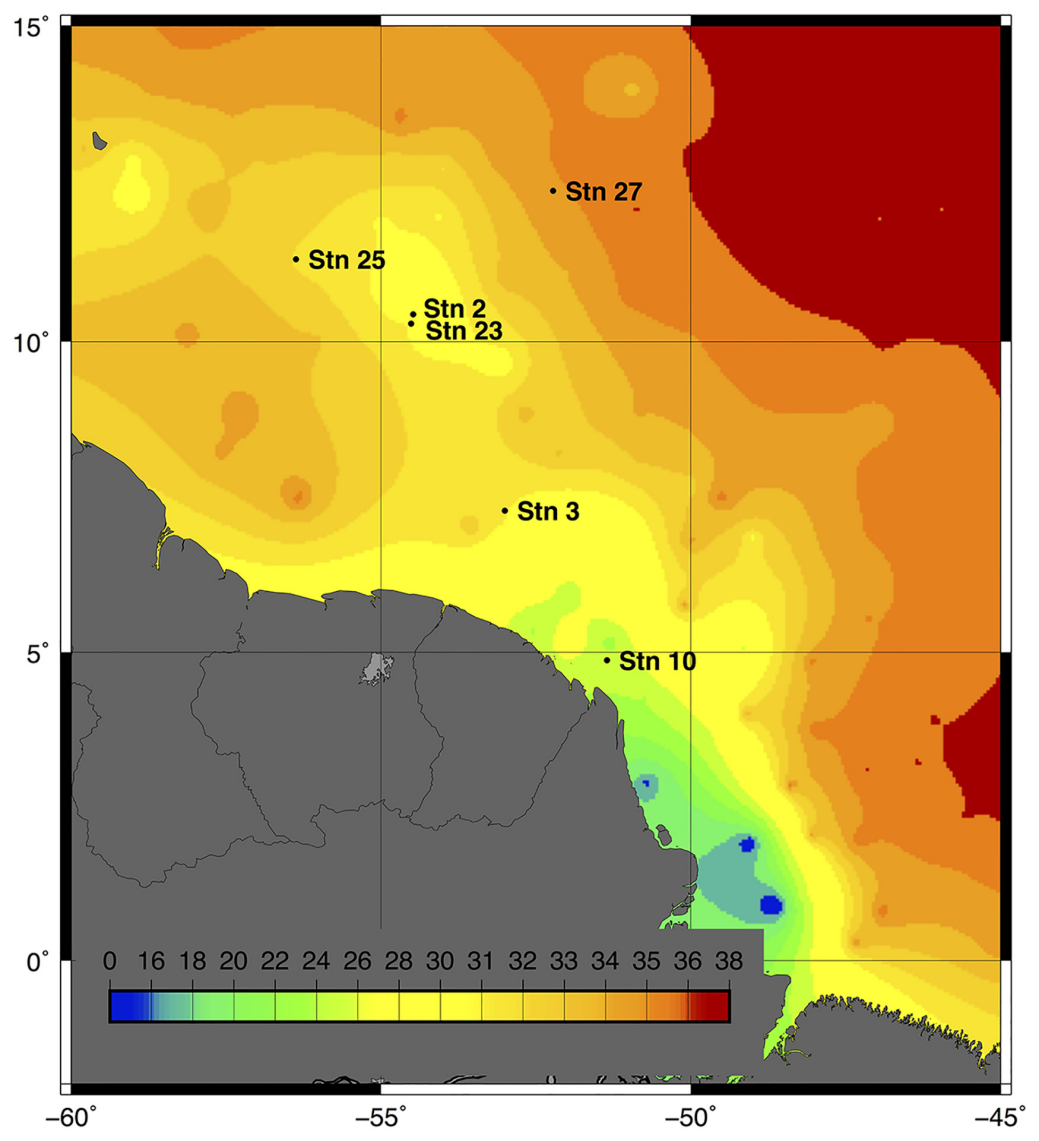
Salinity map of the May/June 2010 Amazon River Plume cruise aboard the RV Knorr. Salinity (PSU) from the underway system along the ship track was augmented with National Oceanographic Data Center profiles in regions of low coverage then interpolated and contoured.

The eukaryotic population in the >2.0 μm size fractioned surface water (156 μm pre-filtered) was examined at six unique stations (duplicate samples at each location, total of 12 samples). Of the 6 stations we sampled (Fig 1; Table 1), one (Sta. 10) was in the coastal plume on the shallow continental shelf (lowest salinity of 21.7 PSU), two (Sta. 3 and 23) were in the near outer plume (salinity of 26–30 PSU), two (Sta. 2 and 25) were in the mesohaline zone (salinity of 31) expected to favor DDAs, and one (Sta. 27) was in the far outer plume or oceanic zone (salinity >35). Stations 2 (surface salinity 31.4 PSU) and 23 (surface salinity 26.2 PSU) were sampled geographically near each other, but about three weeks apart, illustrating the dynamic nature of the plume by their salinity difference.

**Table 1:** Metadata for stations sampled in the ARP. Measurements taken in conjunction with the metatranscriptomes are listed here. Asterisks highlight where concentration of the variable was below limit of detection.

Station	Latitude	Longitude	Date Sampled	Salinity	Sea Surface Temperature (°C)	Mean Phosphate (μM)	Mean Silica (μM)	Mean NO3 + NO2	CTD Beam Transmittance	DIC	Diatom Microscope Count (cells/L)	Hemialius Microscope Count (cells/L)	Chl (μg/L)
**2**	10.29 N	54.51 W	5/25/10	31.362	28.87	0.11	7.34	0*	89.20	1802	165,935	164,531	3.251
**3**	7.29 N	53.00 W	5/26/10	30.086	28.96	0.19	17.03	0*	92.37	1846	125,962	1,049	0.580
**10**	4.88 N	51.36 W	6/5/10	21.721	29.61	0.39	38.48	0.188	46.12	1372	6,940,996	0*	36.107
**23**	10.62 N	54.40 W	6/16/10	26.177	29.51	0.37	26.33	0*	93.81	1575	26,322	3,210	0.150
**25**	11.32 N	56.43 W	6/18/10	31.310	29.43	0*	2.81	0*	85.83	1774	372,113	371,000	5.250
**27**	12.41 N	52.22 W	6/21/10	35.311	28.65	0.27	1.23	0*	95.03	1992	1,608	402	0.129

Transcript counts were normalized by sample-size. Duplicates at each station were analyzed to determine variation in gene expression over distance and time sampled. A direct comparison of normalized transcript counts between duplicates were very similar (R = 0.922) over all the stations (S1 Fig). The average difference between duplicate transcript counts was 11.43%. The similarity in duplicates over space and time suggests that expression levels of microbial eukaryotic communities can be stable over distances of up to 2.45 km (station 23) and time intervals of up to 2 h (station 10), when environmental factors such as salinity, temperature, illumination, and nutrient concentrations are also similar. This is an important result, because previous work has only demonstrated a stability in transcription abundance in environmental samples within eight minutes [42]. Had there been large transcriptional differences in the 31 examined genes, without commensurate environmental change, these data would be difficult to link to biogeochemical cycling or model development. These data suggest that, for at least a subset of genes, transcripts that relate to the local environmental conditions can be used to assess the role of the eukaryotic microbial communities in biogeochemical cycles. Replicates also have been shown to provide greater transcript detection power compared to an increased sequence depth, highlighting the importance of replicates in measuring differences in transcript activity among genes with low mRNA copy number [43]. Duplicate sample values are presented as the average of the duplicates in the ensuing analysis described below.

**Fig 2:**
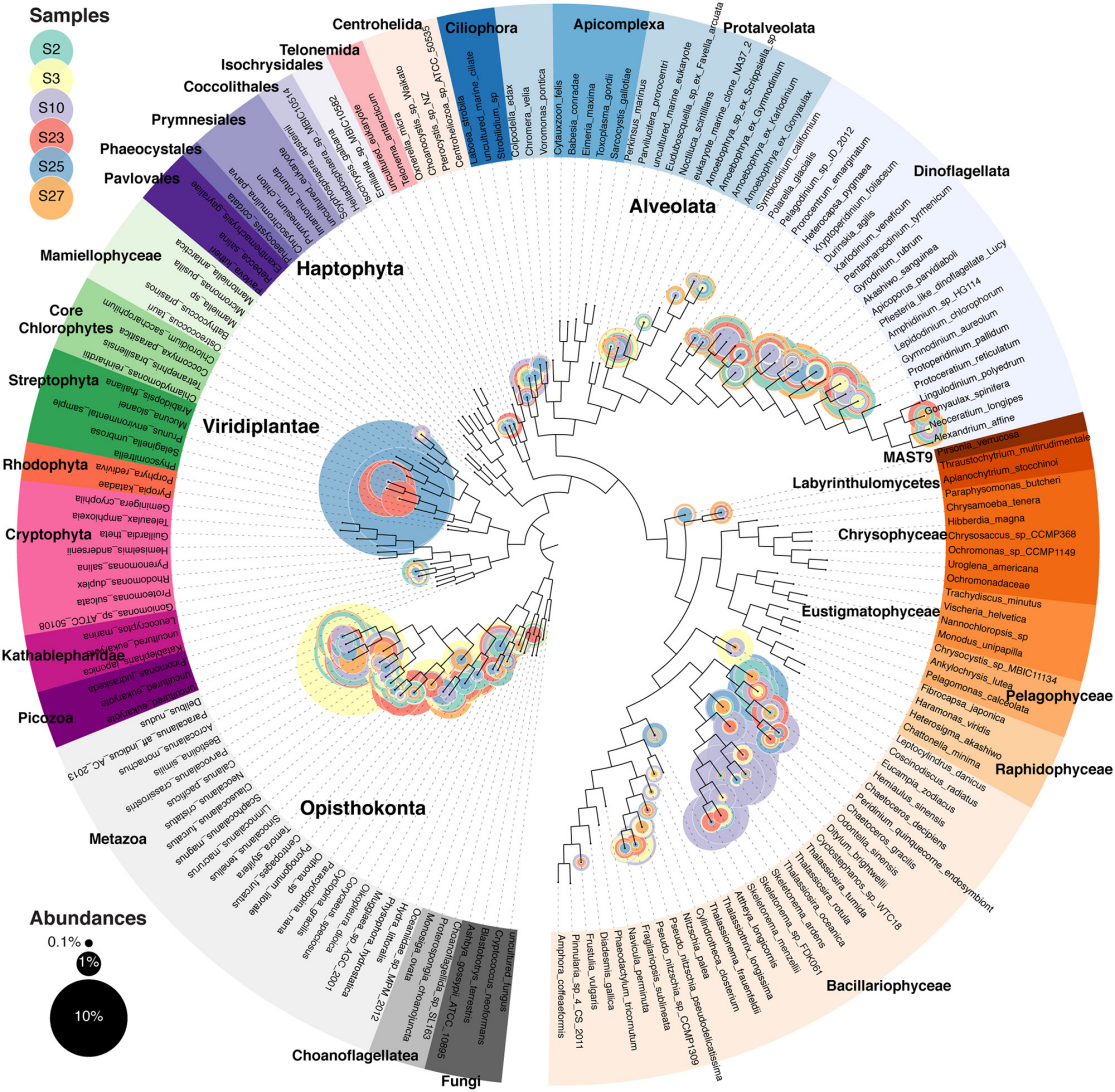
Metagenomic profiling of 18S rDNA for all six stations. Nuclear small subunit 18S rDNA maximum likelihood tree with the placement of environmental sequences. Each circle represents one branch, and sizes are proportional to the normalized taxonomic abundances. Individual trees for each station can be found in Supplemental Materials (S2, S3, S4, S5, S6 and S7 Figs).

Eukaryotic community structure varied across the six stations (Fig 2, S2, S3, S4, S5, S6 and S7 Figs). Despite the 156 μm prefilter, larger cells were still observed due to the filtering process breaking chains and lysing cells. Over all six stations, the 18S rDNA recovered was 19.2% ± 2.5% diatom, 18.04% ± 4.3% dinoflagellate, and 38.50% ± 3.8% metazoan origin. Since the 18S rDNA gene has variable copy numbers per genome, relative abundance using 18S results do not represent absolute community abundance [44]. Station 10, with the lowest surface salinity and the only detectible dissolved inorganic nitrogen concentration (0.18 μmol L^-1^), contained a large diatom bloom consisting principally of the centric diatoms *Thalassiosira* and *Cyclostephanos* (S5 Fig), according to best Blastn hit. The thick patches of Chl *a* in conjunction with elevated inorganic nitrogen levels agrees that this station had a large mixed population of diatoms [5]. *Hemiaulus sp*., a diatom, which often forms a DDA with the endosymbiotic cyanobacteria *Richelia intracellularis*, was found in the 18S rDNA sequences at stations 2, 10 and 25. *R*. *intracellularis* was also confirmed in these stations due to the elevated concentrations of PE-2 phycobilipigments [5]. This *Hemiaulus sp*. population was confirmed by epifluorescence microscopy at only stations 2 and 25 (Table 1), suggesting that the limit of detection is lower for the metagenome and that low abundances of DDAs may occur even when inorganic nitrogen is available. Station 25 had more abundant *Hemiaulus* cells (3.71 x 10^5^ cells L^-1^) than station 2 (1.64 x 10^5^ cells L^-1^). Epifluorescence microscopy observations indicated a healthy DDA community at station 2, as the DDAs had brightly fluorescent chloroplasts and formed long chains, while DDA chains from station 25 were short and/or broken and had weak fluorescence, suggesting bloom senescence.

Low levels of *Hemiaulus* were also found at stations 3 and 23, and the phytoplankton communities displayed the salinity transition from station 10 to stations 2 and 25. One exception to this is that the diatom *Leptocylindrus danicus* (a typically estuarine diatom)[45] was numerous as station 3 and *Chaetocerous decipiens* (a typically coastal diatom)[46] was most abundant at station 23. These intermediate plume stations contained the largest metazoan population, largely consisting of *Bestiolina similis*, a copepod known to be at high abundance in nearshore waters [47] (S3 and S5 Figs). Since this organism should have been captured in the prefiltering process, this signal is likely eggs or nauplii larvae to that passed through [48].

The 18S rDNA data at Station 25 also suggest a large embryophyte (land plants) signature (51.09% of 18S rDNA). The sequences align weakly with the *Mucuna* genus, which consists of climbing vine and shrub vascular plant. Rafts of terrestrial plant debris were occasionally observed in the plume, so it is possible we collected some of that material. Chlorophytes have very similar 18S rDNA sequences, however, and due to short sequence length, these “embryophytes” may have instead represented an uncultured chlorophyta.

Oceanic station 27 was the most oligotrophic station, with the highest surface salinity (35.3), undetectable dissolved inorganic nitrogen, and the lowest eukaryotic phytoplankton cell counts (Table 1). Here, the DDAs observed by epifluorescence microscopy had empty frustules. The 18S phylogenetic data suggests this station was the station with the largest proportion of dinoflagellates, with *Gyrodinium rubrum* and *Apicoporus parvidiaboli* as the most abundant species. The dinoflagellate population has been reported to exist in much smaller numbers throughout the plume compared to the other phytoplankton groups [5].

### Expression of Key Biogeochemically-Relevant Genes

Station 10 had the highest number of total transcripts for 16 of 31 genes (Table 2), and also had the highest chlorophyll *a* concentration and the only measurable dissolved inorganic nitrogen (Table 1). This outcome is likely a function of the thriving coastal diatom bloom fueled by riverine nutrients (e.g., nitrate, phosphate, silicate). At all the stations, glyceraldehyde-3-phosphate dehydrogenase (*GADPH*, carbon heterotrophy) was the most highly expressed in terms of transcript number of all 31 genes. At Station 10, the eukaryotic nitrate transporter (*NRT*) was also expressed, along with two carbon autotrophy genes (delta carbonic anhydrase and transketolase). At stations 3 and 23, *NRT* was less abundant than at Station 10 and the ammonium transporter (*amtB*) increased in its relative importance. Stations 2 and 25 were unique in their high expression of photosystem II D1 protein (*psbA*) and a silicon transporter (*SIT*). Two of the three most abundant genes at station 27 were acetoacetyl-CoA reductase and polyhydroxybutyrate biosynthesis (consisting of both beta-ketothiolase and NADPH-linked acetoacetyl coenzyme A reductase), and the relative contribution of autotrophic genes were minimal, signifying that at this nutrient-poor station, carbon heterotrophy was dominating.

**Table 2:** Sample size-normalized gene counts for the 31 biogeochemically-relevant genes. Values are the average of the duplicate samples, per 10 million sequences. Bolded/underlined numbers highlight the highest expression for that gene.

Gene Abbreviation	Gene Name	Station 2	Station 3	Station 10	Station 23	Station 25	Station 27
**rbcL_IB**	RuBisCO form IB	7	**46**	24	16	8	1
**rbcL_ID**	RuBisCO form ID	24	15	75	2	**27**	1
**psbA**	Photosystem II protein D1	**9005**	350	1487	106	8493	78
**a-CA**	Carbonic anhydrase (alpha)	216	**367**	332	266	179	161
**d-CA**	Carbonic anhydrase (delta)	740	484	**2388**	498	461	466
**tkt**	Transketolase	1117	697	***2082***	269	620	180
**casE**	Chitinase	277	**565**	110	182	385	171
**Chs3p**	Chitin synthase III	2	17	**552**	1	2	0
**bglA**	Beta-glucosidase	**516**	413	313	491	386	345
**GADPH**	Glyceraldehyde-3-phosphate dehydrogenase	12782	6869	**16908**	6043	12237	6931
**GPI**	Glucose-6-phosphate isomerase	229	339	**584**	192	249	231
**metF**	Methylene tetrahydrofolate reductase	**379**	148	175	106	287	130
**phaB**	Acetoacetyl-CoA reductase	1676	1734	**1883**	1258	1478	1141
**phaA**	Beta-ketothiolase	1125	**1294**	898	1079	876	1063
**AA_Permease**	Amino acid permeases	236	188	**536**	100	287	64
**AAP**	Alanine aminopeptidase	106	116	**236**	62	70	54
**LAP**	Leucine aminopeptidase	370	**684**	557	442	282	251
**amtB**	Ammonium transporter	**340**	170	217	211	263	144
**ProAP**	Proline aminopeptidase	213	294	**297**	246	169	144
**UT**	Eukaryotic urea transporter	128	204	**661**	171	96	51
**MetAP**	Methionine aminopeptidase	417	503	**613**	456	363	355
**NAT**	Eukaryotic nitrate transporter	563	212	**7960**	175	325	27
**pitA**	Low affinity phosphate transporter	**484**	72	216	153	182	80
**ppk2**	Polyphosphate kinase 2	82	**112**	66	73	54	102
**cysK**	Cysteine synthetase A	294	353	**447**	278	246	217
**Xsc**	Sulfoacetaldehyde acetyltransferase	63	62	48	**85**	53	42
**SiR-beta**	Sulfite reductase (beta subunit)	118	104	**370**	68	137	37
**SIT**	Silicon transporter family	**2046**	400	1053	280	1666	215
**pdxH**	Pyridoxamine 5'-phosphate oxidase	89	74	29	45	109	**199**
**pdxK**	Pyridoxinal (pyridoxine, vitamin B6) kinase	**30**	10	19	9	10	22
**thiC**	Phosphomethylpyrimidine synthase	1	2	**61**	0	0	0

**Fig 3:**
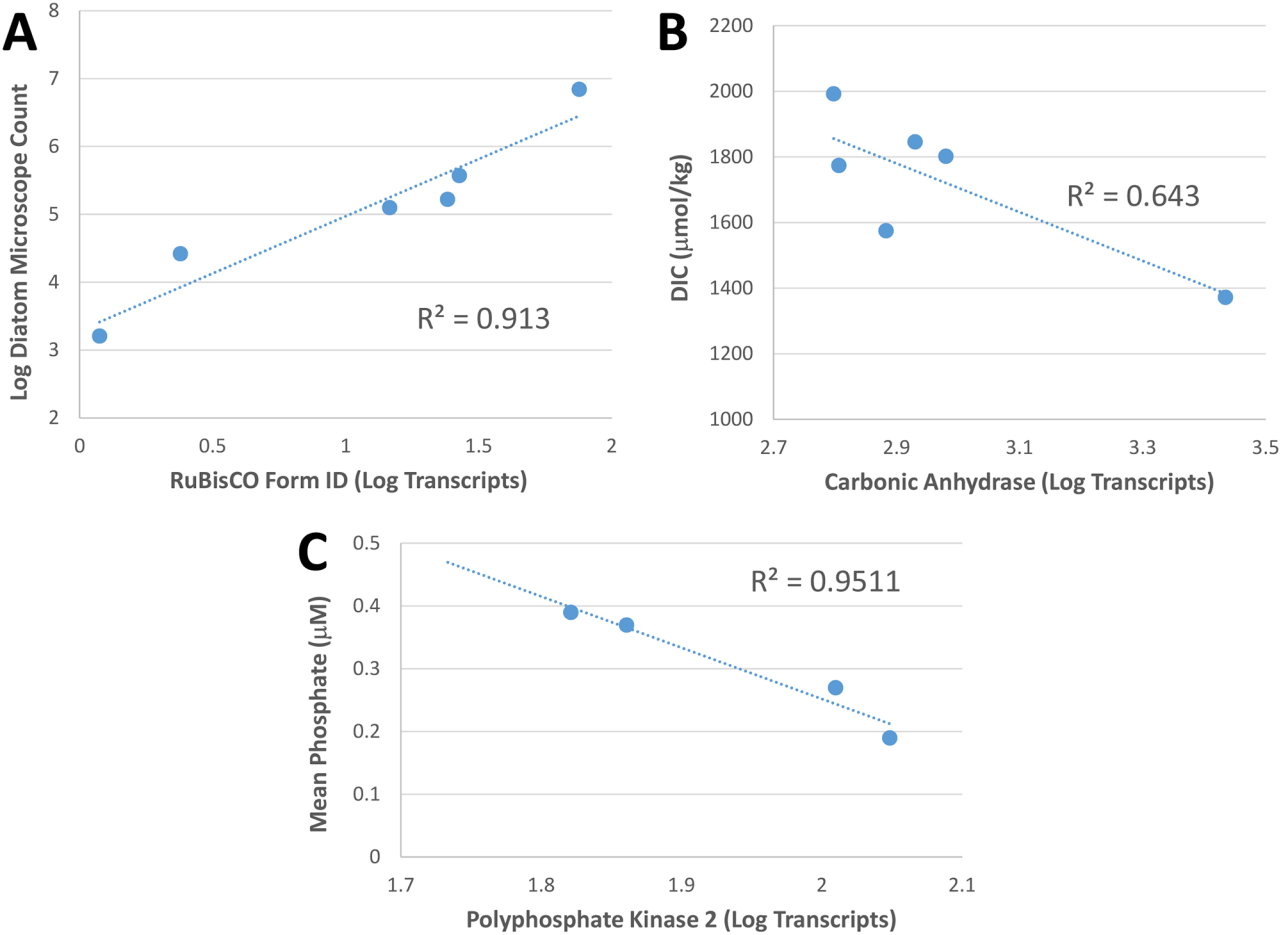
Transcriptomic versus biogeochemical data. Panel A: The correlation between diatom microscope counts and log RuBisCO Form ID transcripts counts. Panel B: The inverse relationship of carbonic anhydrase transcript abundance to DIC concentration. Panel C: The inverse relationship between polyphosphate kinase transcript abundance and phosphate concentration. Station 2 and 25 had little or no phosphate, due to the diatom bloom, however *ppk* was not upregulated.

Ribulose-1,5-bisphosphate carboxylase/oxygenase (RuBisCO) is a key enzyme for carbon fixation, and the different forms of RuBisCO yield important information on the carbon fixing populations present. The large subunit of ribulose-1,5-bisphosphate carboxylase/oxygenase (*rbcL*) is a gene found in the chloroplast of many phytoplankton, where transcripts are not usually poly(A)-tailed. The poly(A)-tailed *rbcL* transcripts found are likely the result of post transcriptional modification, where chloroplast transcripts are polyadenylated to accelerate exoribonucleolytic degradation [49]. Form IB *rbcL* from Streptophyta, Chlorophyta, and Euglenozoa [50] was more strongly expressed at stations 3, 10 and 23, whereas Form ID, found in diatoms [50], and was most abundant at stations 2, 10 and 25, where diatom blooms were observed. As might be expected, a correlation occurred between diatom abundance (from microscope counts) and log RuBisCO form ID transcript abundance (R = 0.956; Fig 3A).

Photosystem II protein D1 (*psbA*) is responsible for binding chlorophylls, quinones and metal ligands in the photosystem II reaction center. This protein undergoes rapid, light-dependent turnover (the “photosystem II repair cycle”). Under illumination *psbA* degrades, and repair or re-synthesis of the D1 protein is necessary to limit the accumulation of photodamaged photosystem II proteins [51]. Photodegradation of the D1 protein occurs under any illumination at a rate roughly proportional to the transfer of excitation energy to the reaction center [51]. The eukaryotes at DDA stations had higher concentrations of *psbA* transcripts than other stations. At the mesohaline DDA stations, there was less chromophoric dissolved organic matter; with the high CDOM from the river being diluted by low-CDOM oceanic water [52, 53], increasing light transmittance (Table 1). The upregulation of *psbA* may be a repair mechanism for photosynthetic blooms in clearer waters to combat high incident irradiance penetration, especially for a rapidly photosynthesizing community. Bloom senescence at station 25 may explain the slightly lower *psbA* transcript counts recovered from that environment. We might expect to see it also expressed well at station 27, but we did not; likely this is because there are so few diatoms there.

Carbonic anhydrase (*CA*) is responsible for the interconversion of bicarbonate and carbon dioxide and is a critical component of carbon concentrating mechanisms of photoautotrophs. Chlorophytes contains α-*CA* [54], which was most highly expressed at stations 3 and 10, where *rbcL* Form IB was also highly expressed signifying chlorophyta populations. δ-*CA*, which is commonly found in diatoms [55], was highly expressed at stations 2 and 10 relative to the other stations, and this same pattern was detected with *rbcL* Form ID, which is the form used in haptophytes, rhodophyta and heterokonts (including diatoms) [50]. There was a strong inverse correlation between DIC concentration and total *CA* transcripts (R = 0.802, Fig 3B). As CO_2_ becomes depleted due to high rates of photosynthesis, organisms expressing *CA* genes will be more successful in supplying CO_2_ to their carbon fixation machinery. Transcript abundance for transketolase (*tkt*), part of the reductive Calvin-Benson-Bassham Cycle, showed a similar, although weaker, inverse relationship with DIC (R = 0.58).

Available nitrogen plays a large role in determining the abundance and composition of marine phytoplankton populations globally [8]. Since ammonium requires less energy to assimilate than other forms of nitrogen in seawater, it often is the preferred nitrogen source for phytoplankton growth. Nitrate is usually used by phytoplankton if other forms of reduced nitrogen (ammonium, urea) are absent and there is an appreciable amount of nitrate to support high growth rates [56, 57]. A large phytoplankton bloom made up of chain-forming diatoms, was present at Station 10, where eukaryotic nitrate transport (*NRT*) expression was highest. Station 10 was also the only station with measurable dissolved nitrogen (Table 1). If an appreciable amount of ammonium is available, it strongly downregulates *NRT* expression [58], signifying that ammonium concentrations (not measured) were not high enough to support this diatom bloom. A phylogenetic analysis of expressed *NRT* genes revealed that most of the transcription was carried out by the diatom *Chaetoceros* and the chlorophyte *Micromonas* (S8 Fig), which were also observed to be highly abundant through epifluorescent microscopy. The lack of detectable nitrate at the other stations explains low abundance of *NRT* transcripts at these stations since slower growth rates can be supported by ammonia utilization. Cell-surface ammonium transporter (*amtB*) expression levels were highest at the DDA stations, where the extracellular endosymbionts (residing between the plasmalemma and silica wall) fix nitrogen and transfer it to diatoms [7], possibly in the form of ammonium [58]. Urea occurs frequently in nature as a result of release of nitrogenous wastes and is considered “recycled N”. Urease, synthesized by almost all organisms, is used to hydrolyze urea to carbon dioxide and ammonia. This conversion provides an important nitrogen source in otherwise nitrogen-limited environments. However, the *Richelia* and *Calothrix* symbionts in the DDAs lack both urease and urea transporters [59]. We observed that the urea transporter (*UT*) showed the highest transcript abundance at stations closest to the mouth of the river, most likely caused by the terrestrial input of urea.

The low affinity phosphate transporter (*pitA*) is highly expressed at station 2, consistent with a thriving phytoplankton bloom with available inorganic phosphate (P_i_). *PitA* is expressed when P_i_ is plentiful, but when concentrations of P_i_ are low, the high affinity phosphate transporter is induced instead [60]. Station 25 was the only station without detectable P_i_, and consequently had low expression of *pitA*. Polyphosphate kinase (*ppk*) catalyzes the reversible transfer of the terminal phosphate of ATP to form a long chain polyphosphate [61]. A biochemical characterization of *ppk* in eukaryotes has not been reported, and with the reaction being reversible, interpreting the differing levels of expression is problematic. Nonetheless, with the removal of data from the DDA stations 2 and 25, there was a correlation of *ppk* transcript abundance and phosphate concentration (R = 0.975, Fig 3C). These stations have little or no measureable phosphate and are likely using some other method for acquiring phosphorus. This strong correlation at the other four stations suggests that cleavage of a terminal phosphate from a polyphosphate may be a scavenging technique for microbial eukaryotes under phosphate depleted (impoverished) conditions [62].

Silica transport is required for the synthesis of diatom frustules. Diatoms need to maintain an intracellular concentration of soluble silica sufficient for complete cell wall synthesis, which generally occurs within an hour [63]. Regulation of synthesis is necessary to prevent polymerization prior to deposition [63]. Consequently, all silica transporter proteins (*SIT*) are induced at once, just prior to the maximum incorporation of silica into the cell wall [63]. High expression of *SIT* signifies a rapidly dividing diatom population, as observed at the diatom abundant stations 2, 10 and 25. Station 27, with the smallest diatom population and the lowest silica concentration had the lowest *SIT* expression.

The abundance of chitinase (*casE*) and chitin synthase (*Chs3p*) transcripts together can account for the fate of chitin in the microeukaryotes in the ARP. Chitin synthase can be found in both copepods and diatoms, however since copepods were only minimally represented in station 10, due to the prefilter, the measured chitin synthase expression is likely from diatoms containing this gene, such as *Thalassiosira* and *Cyclotella* [64, 65]. Diatoms produce chitin to decrease sinking rates by increasing buoyancy with extruding chitin fibers from the frustule pores [65, 66]. These chitin fibers can account for up to 40% of the total cell biomass [67]. Another role for chitin in diatoms is as a substitute constituent of cell walls during long-term silicic acid starvation [68]. However this is unlikely to be the case in our samples because the station with the highest *Chs3p* expression (Station 10) had the highest silica concentration (39.30 μmol L^-1^). Some diatoms at station 10 were very large (>150 μm), and likely were using *Chs3p* to decrease sinking rates. Chitinase expression was highest at Station 3, and then also relatively high in stations 2 and 25, possibly in response to metabolizing the chitin produced by the larger diatoms.

### Patterns of Gene Expression

Our hypothesis is that relative magnitude of transcription of certain important biogeochemical gene functions in seawater samples often reflects or correlates with biogeochemical processes taking place at the point and time of sampling, and these data support this hypothesis. For example, high expression of *psbA* co-occurred in surface phytoplankton populations in clear water (Stations 2 and 25). Furthermore both *SIT* and Form ID *rbcL* are diagnostic of healthy diatom populations. High expression of *NRT* signified that nitrate was utalized to fuel high growth rates, as observed at station 10. Using a combination of these predictable differences of transcript abundances, or ‘patterns of gene expression’, biogeochemical processes can be related to the transcripts observed.

**Fig 4:**
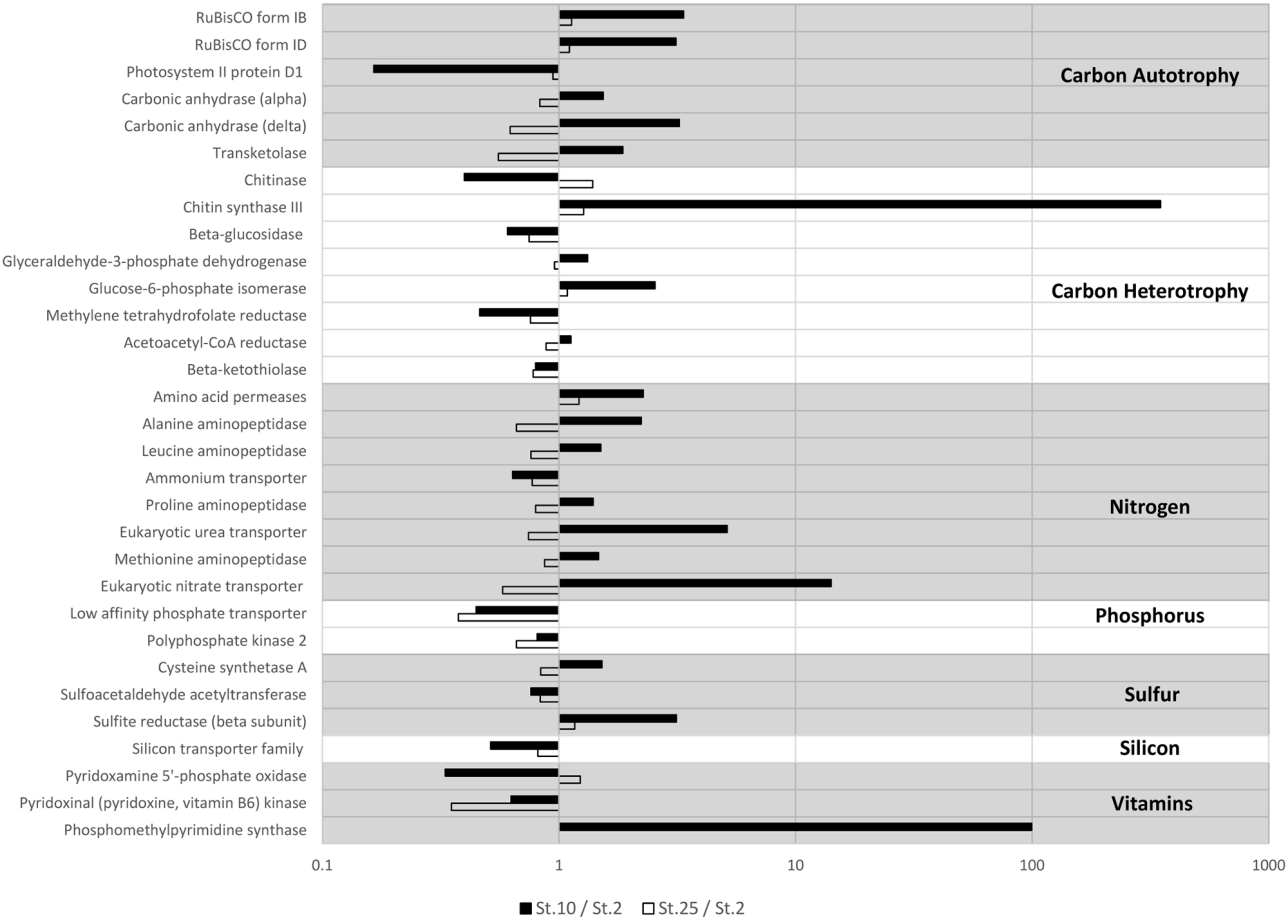
Ratios of transcript abundance at stations 10:2 (black bars) and 25:2 (white bars). Station 10 has very high levels of eukaryotic nitrate transporter as well as chitin synthase compared to station 2. Note log scale. Stations 2 and 25 perform similar functions in the ARP. Thus the plot of the ratio of Station 25: Station 2 has smaller values than the ratio of stations 10 and 2.

Direct comparisons by ratios between the patterns of gene expression at the three stations which were abundant in diatoms illustrates how these data reveal the deviations between the stations (Fig 4). Station 10 shows high expression of chitin synthase III, perhaps for buoyancy or defense [69], and *NRT* due to the nitrate availability. Despite being sampled 238.3 km and 25 days apart, the DDA stations 2 and 25 show very little deviation between transcript counts, with the average difference between transcript abundance being 9.33%. This evidence supports the notion that patterns of gene expression are stable in similar microbial eukaryote communities living in similar environments and thus suggests that the biogeochemistry and microbial communities are intricately linked.

## Conclusions

This study demonstrates that metatranscriptomic analysis of 31 pre-selected biogeochemically-relevant genes allowed for a reliable analysis of eukaryotic planktonic communities and their physiological status in the ARP. A stability in patterns of gene expression of similar planktonic communities over space and time was demonstrated, allowing for better resolution through replicates. Phylogenetic information from 18S rDNA enabled taxa to be assigned to the short length transcript sequences collected from these environments and transcription was related to environmental conditions, supporting that a metatranscriptomic study can be used to describe the biogeochemistry. This study showed that DDA blooms are capable of upregulating expression for their photosystem II D1 protein and in acquiring silica. The small differences between their expression at exponential growth phase and senescing populations can be further explored in culture. In lower salinity, non-DDA diatom blooms, nitrate transporters are activated to use nitrate to support their high growth rates, and carbonic anhydrase (carbon concentrating mechanism) allowing these diatoms to thrive in low-CO_2_ waters. Finally, chitin synthase was hypothesized to be a mechanism used by diatoms in lower-salinity plume waters to decrease their sinking rates, as light is also limiting in these young plume waters. These results, in conjunction with ongoing modeling efforts, will help us understand the river plume microbial communities in this globally important ecosystem. Future research will involve sampling the ARP in different seasons, comparing the different patterns of gene expression between seasons, and using those data to ground-truth the ecosystem models.

## Supporting Information

S1 Fig Log replicate 1 versus log replicate 2 plot of transcript counts at all six stations. The dotted line represents the 1:1 line of identity. The 186 data points represent the 31 genes measured at 6 stations. The average difference between replicate transcripts was 11.43%.
(TIF)

S2 Fig Metagenomic profiling of 18S rDNA for Station 2. Nuclear small subunit 18S rDNA maximum likelihood tree with the placement of environmental sequences. Circle sizes are proportion to the normalized taxonomic abundances.
(TIF)

S3 Fig Metagenomic profiling of 18S rDNA for Station 3. Nuclear small subunit 18S rDNA maximum likelihood tree with the placement of environmental sequences. Circle sizes are proportion to the normalized taxonomic abundances.
(TIF)

S4 Fig Metagenomic profiling of 18S rDNA for Station 10. Nuclear small subunit 18S rDNA maximum likelihood tree with the placement of environmental sequences. Circle sizes are proportion to the normalized taxonomic abundances.
(TIF)

S5 Fig Metagenomic profiling of 18S rDNA for Station 23. Nuclear small subunit 18S rDNA maximum likelihood tree with the placement of environmental sequences. Circle sizes are proportion to the normalized taxonomic abundances.
(TIF)

S6 Fig Metagenomic profiling of 18S rDNA for Station 25. Nuclear small subunit 18S rDNA maximum likelihood tree with the placement of environmental sequences. Circle sizes are proportion to the normalized taxonomic abundances.
(TIF)

S7 Fig Metagenomic profiling of 18S rDNA for Station 27. Nuclear small subunit 18S rDNA maximum likelihood tree with the placement of environmental sequences. Circle sizes are proportion to the normalized taxonomic abundances.
(TIF)

S8 Fig Metatranscriptomic profiling of nitrate transporters (NRT) at the 6 six stations along the ARP. A maximum likelihood tree was used with the placement of metatranscriptomic predicted open-reading frames. Bootstrap support values ≥ 50% are shown. Circle sizes are proportion to the normalized expression levels. Branch lengths are log10-transformed.
(TIF)

S1 Table Sequencing Data. Compiled data of all the sequences obtained and analyzed at the six stations. Duplicate samples were pooled to account for variations in the data that may occur from only taking one sample.
(TIF)

S2 Table Background data for genes analyzed. (TIF)
