# Supplementary figures and images for: Patterns of Transcript Abundance of Eukaryotic Biogeochemically-Relevant Genes in the Amazon River Plume
Source: PLoS One. 2016 Sep 6;11(9):e0160929. doi: 10.1371/journal.pone.0160929 (PMC5012681; doi:10.1371/journal.pone.0160929)

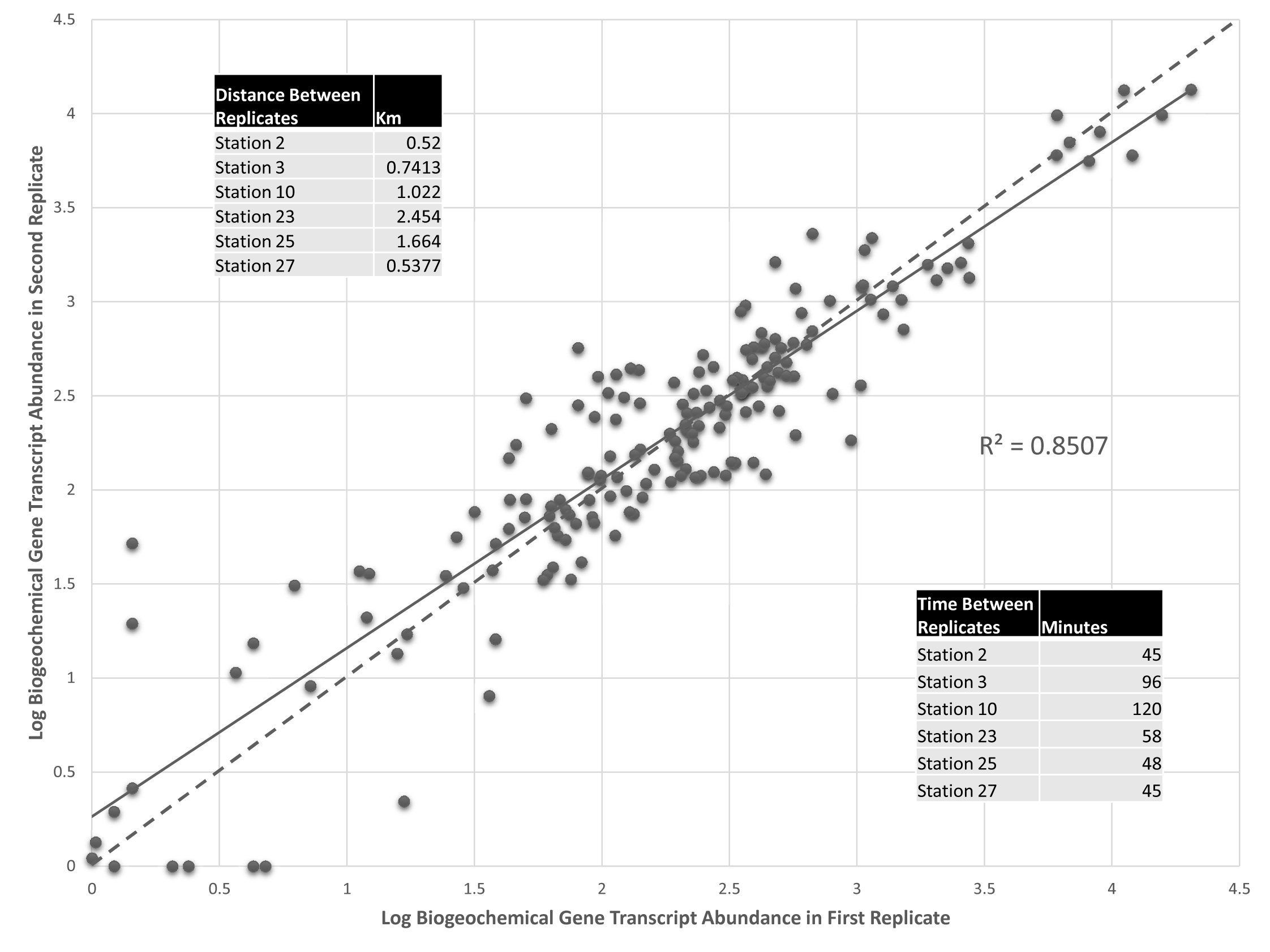

Supplement: S1 Fig — The dotted line represents the 1:1 line of identity. The 186 data points represent the 31 genes measured at 6 stations. The average difference between replicate transcripts was 11.43%. (TIF) [file pone.0160929.s001.tif]

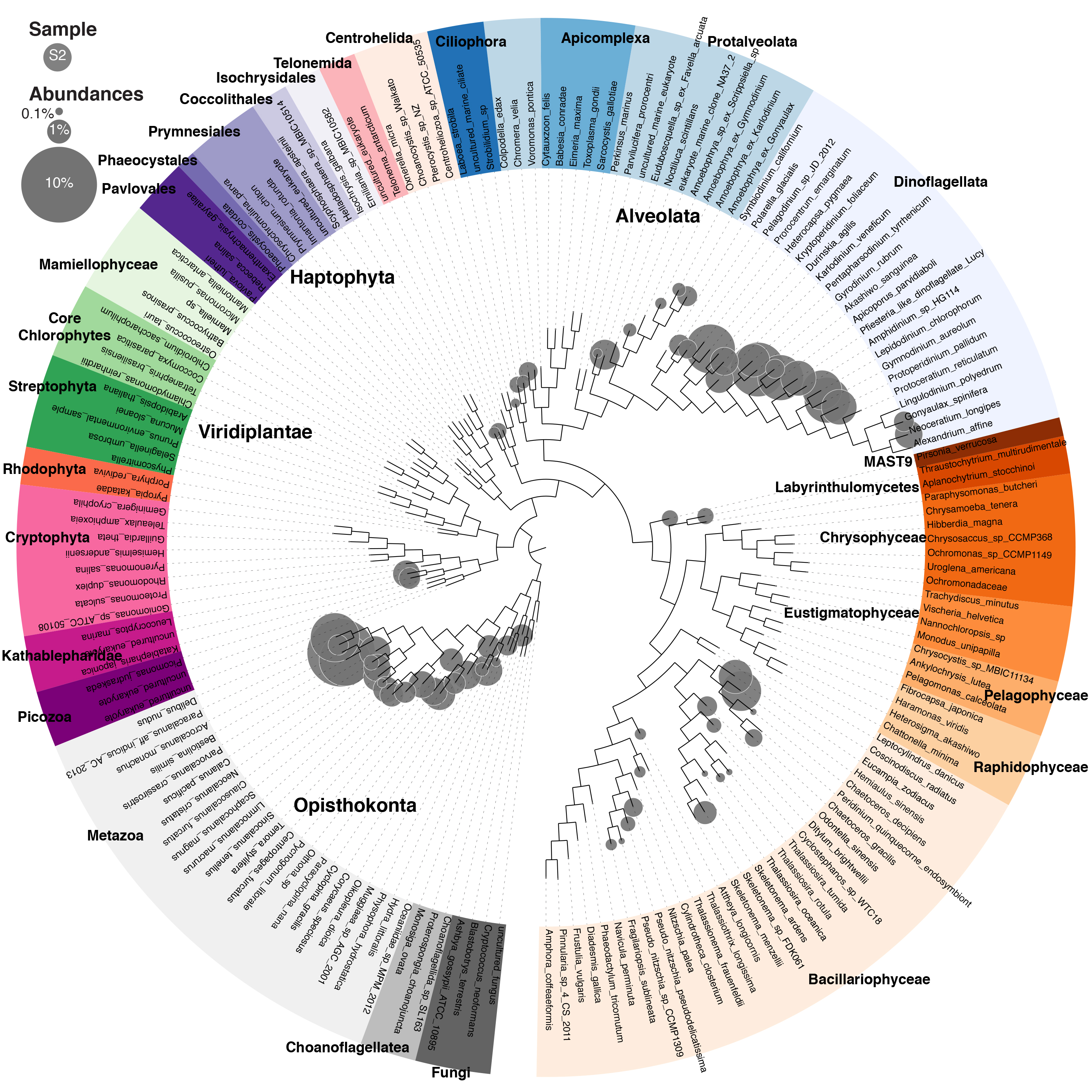

Supplement: S2 Fig — Nuclear small subunit 18S rDNA maximum likelihood tree with the placement of environmental sequences. Circle sizes are proportion to the normalized taxonomic abundances. (TIF) [file pone.0160929.s002.tif]

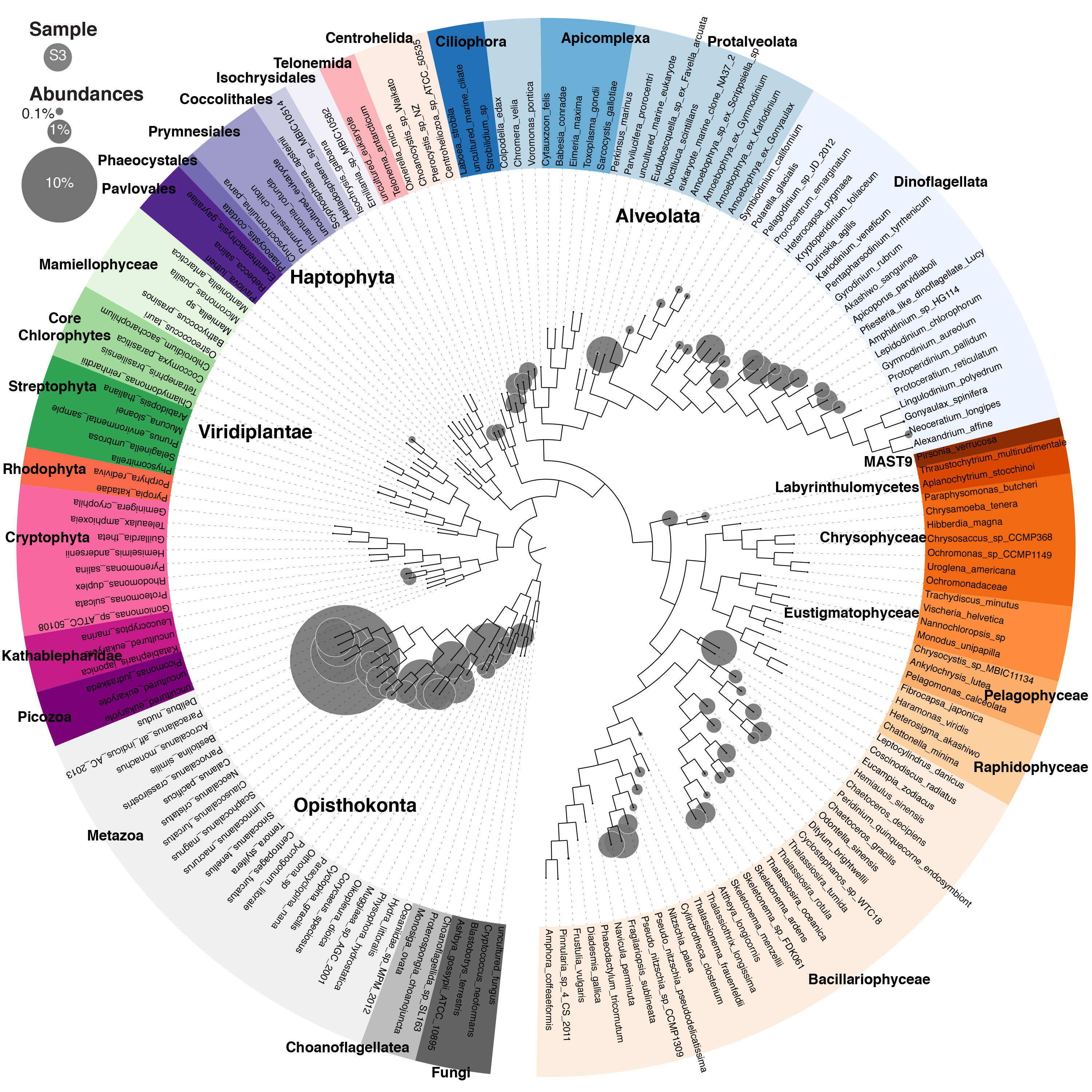

Supplement: S3 Fig — Nuclear small subunit 18S rDNA maximum likelihood tree with the placement of environmental sequences. Circle sizes are proportion to the normalized taxonomic abundances. (TIF) [file pone.0160929.s003.tif]

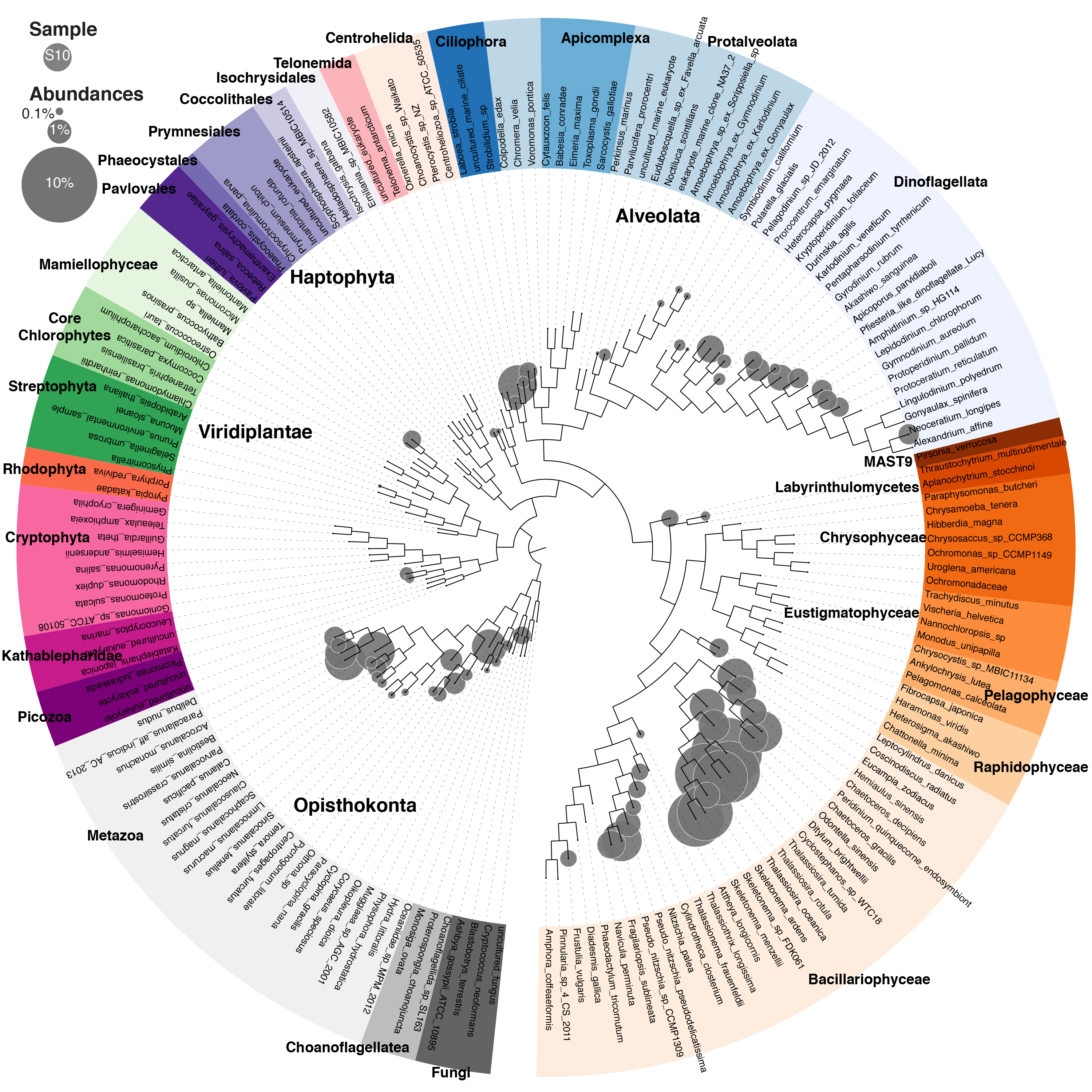

Supplement: S4 Fig — Nuclear small subunit 18S rDNA maximum likelihood tree with the placement of environmental sequences. Circle sizes are proportion to the normalized taxonomic abundances. (TIF) [file pone.0160929.s004.tif]

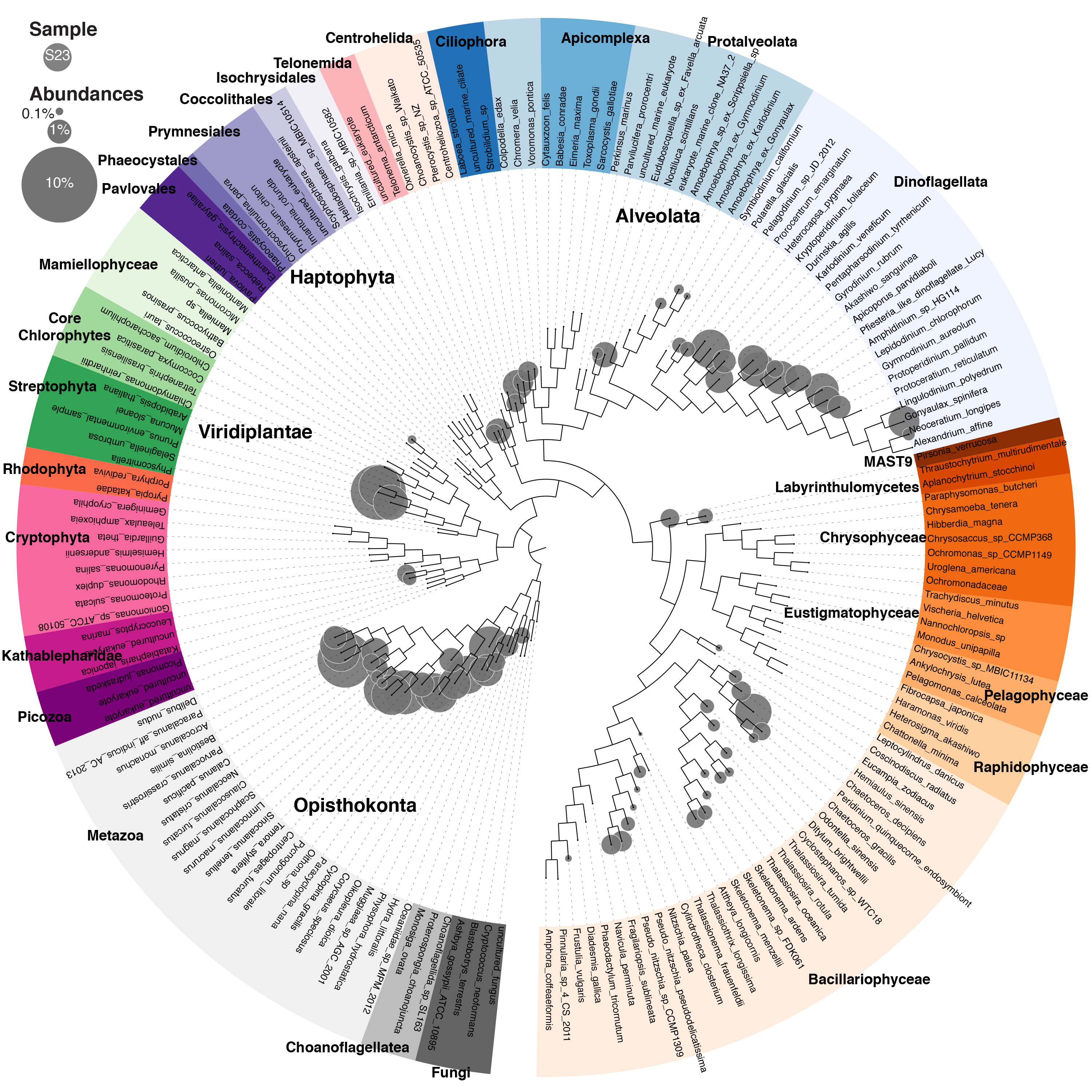

Supplement: S5 Fig — Nuclear small subunit 18S rDNA maximum likelihood tree with the placement of environmental sequences. Circle sizes are proportion to the normalized taxonomic abundances. (TIF) [file pone.0160929.s005.tif]

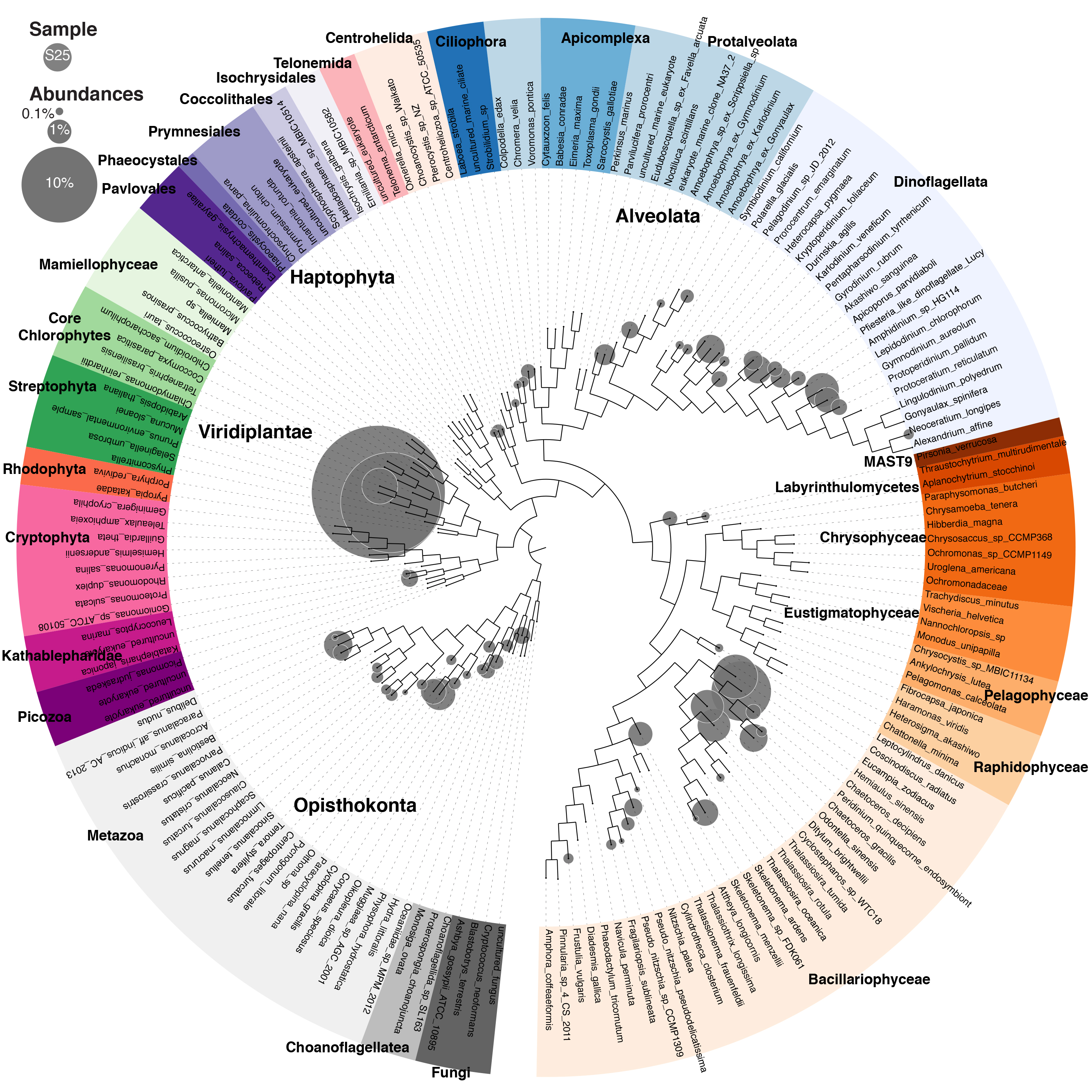

Supplement: S6 Fig — Nuclear small subunit 18S rDNA maximum likelihood tree with the placement of environmental sequences. Circle sizes are proportion to the normalized taxonomic abundances. (TIF) [file pone.0160929.s006.tif]

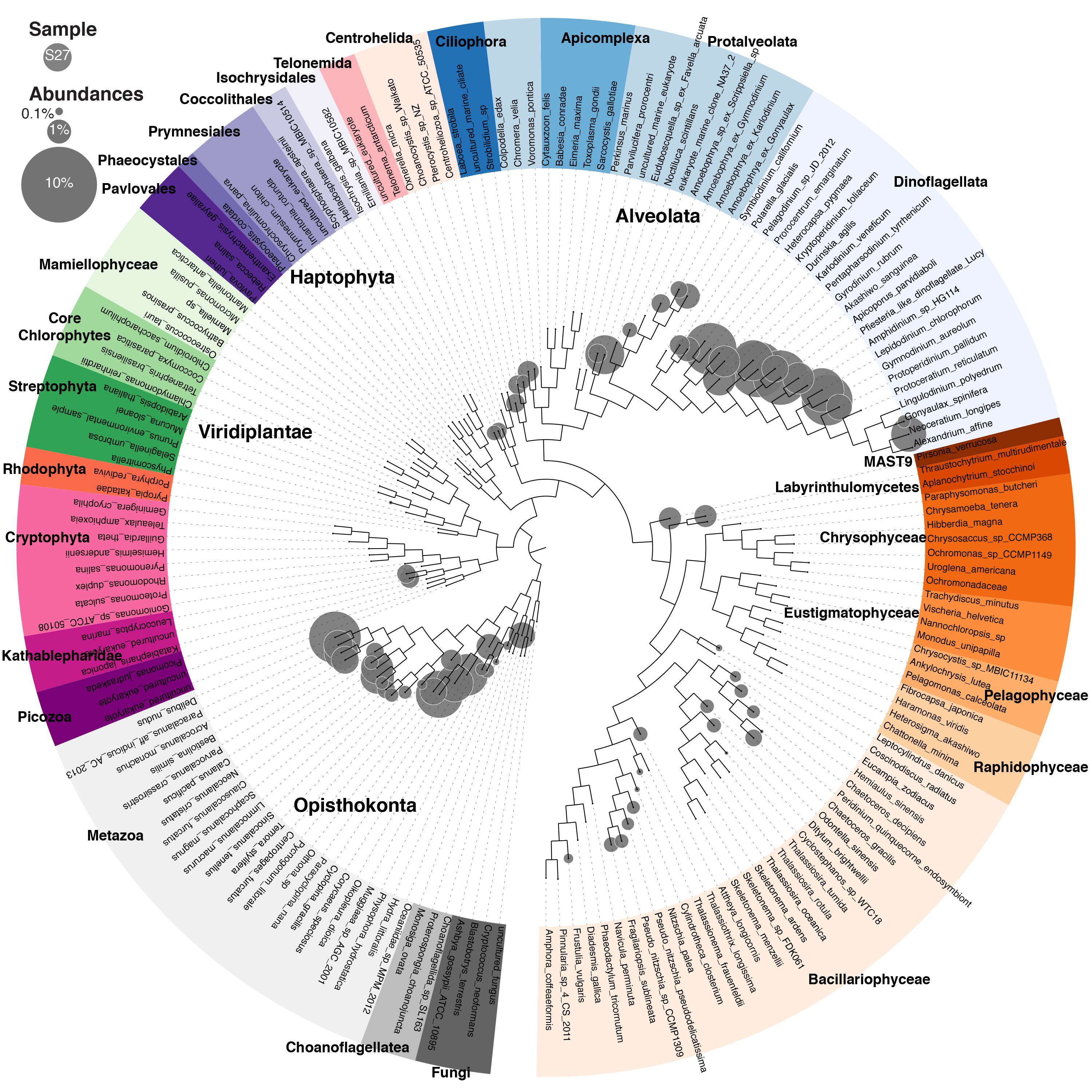

Supplement: S7 Fig — Nuclear small subunit 18S rDNA maximum likelihood tree with the placement of environmental sequences. Circle sizes are proportion to the normalized taxonomic abundances. (TIF) [file pone.0160929.s007.tif]

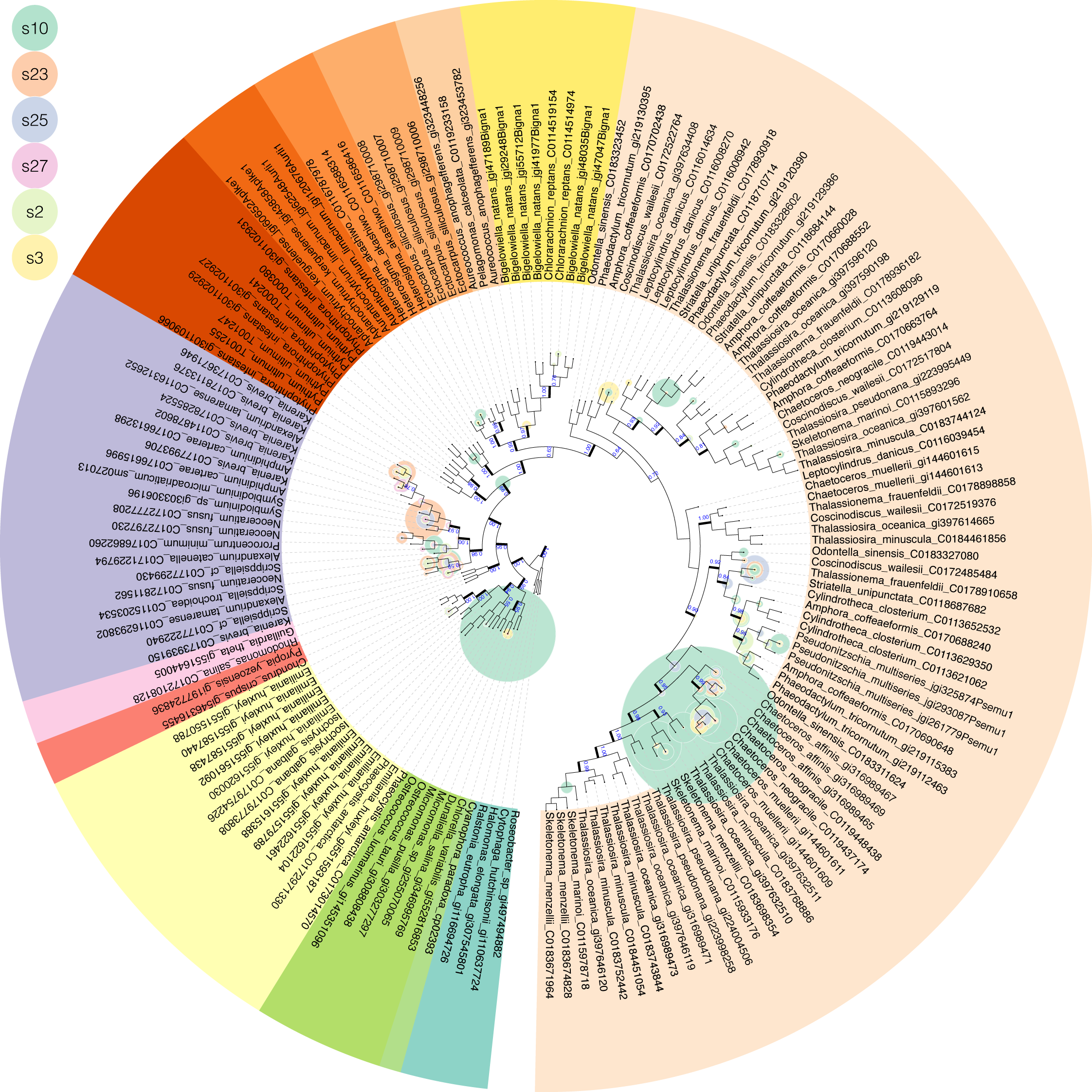

Supplement: S8 Fig — A maximum likelihood tree was used with the placement of metatranscriptomic predicted open-reading frames. Bootstrap support values ≥ 50% are shown. Circle sizes are proportion to the normalized expression levels. Branch lengths are log10-transformed. (TIF) [file pone.0160929.s008.tif]

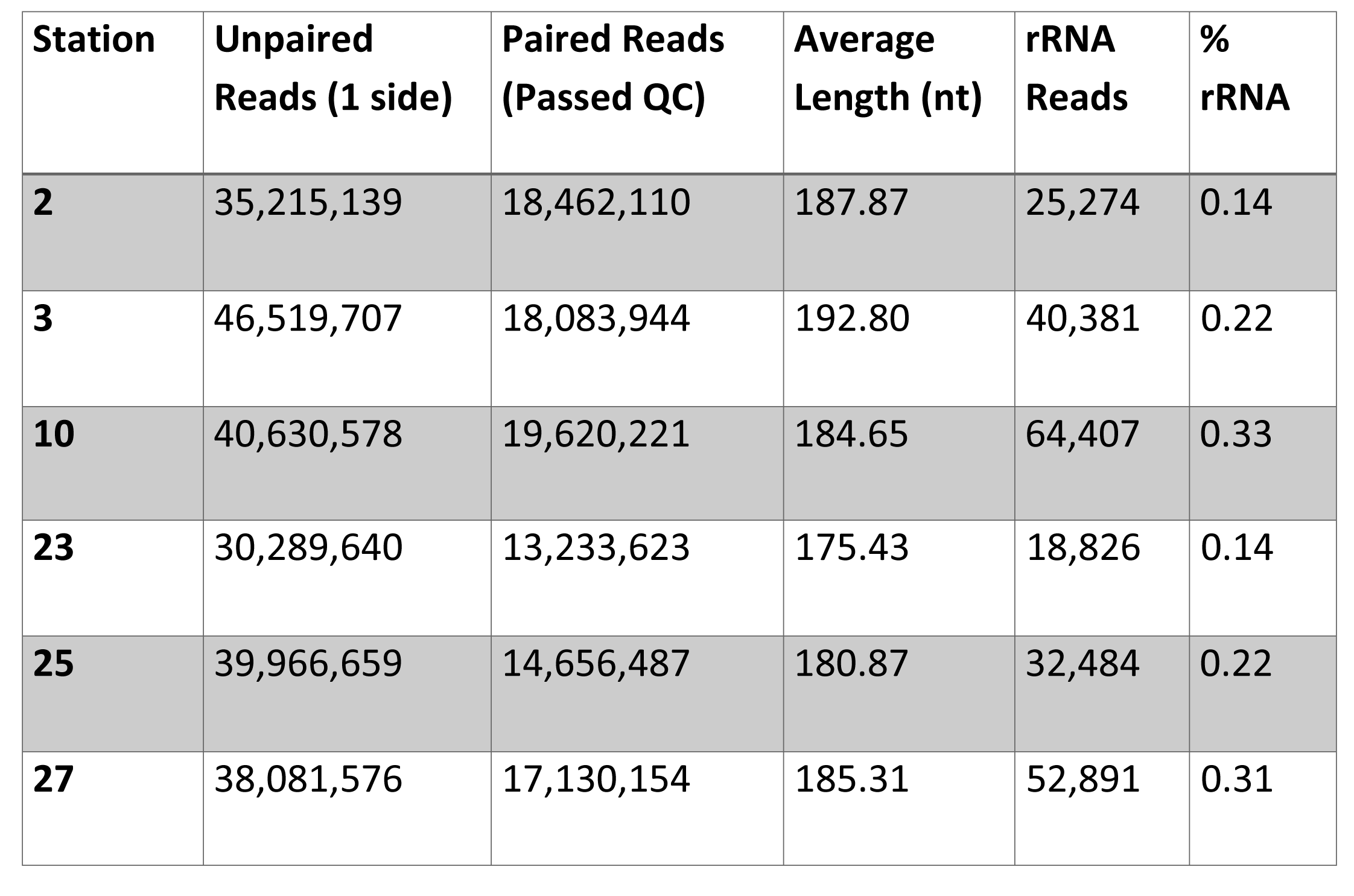

Supplement: S1 Table — Compiled data of all the sequences obtained and analyzed at the six stations. Duplicate samples were pooled to account for variations in the data that may occur from only taking one sample. (TIF) [file pone.0160929.s009.tif]

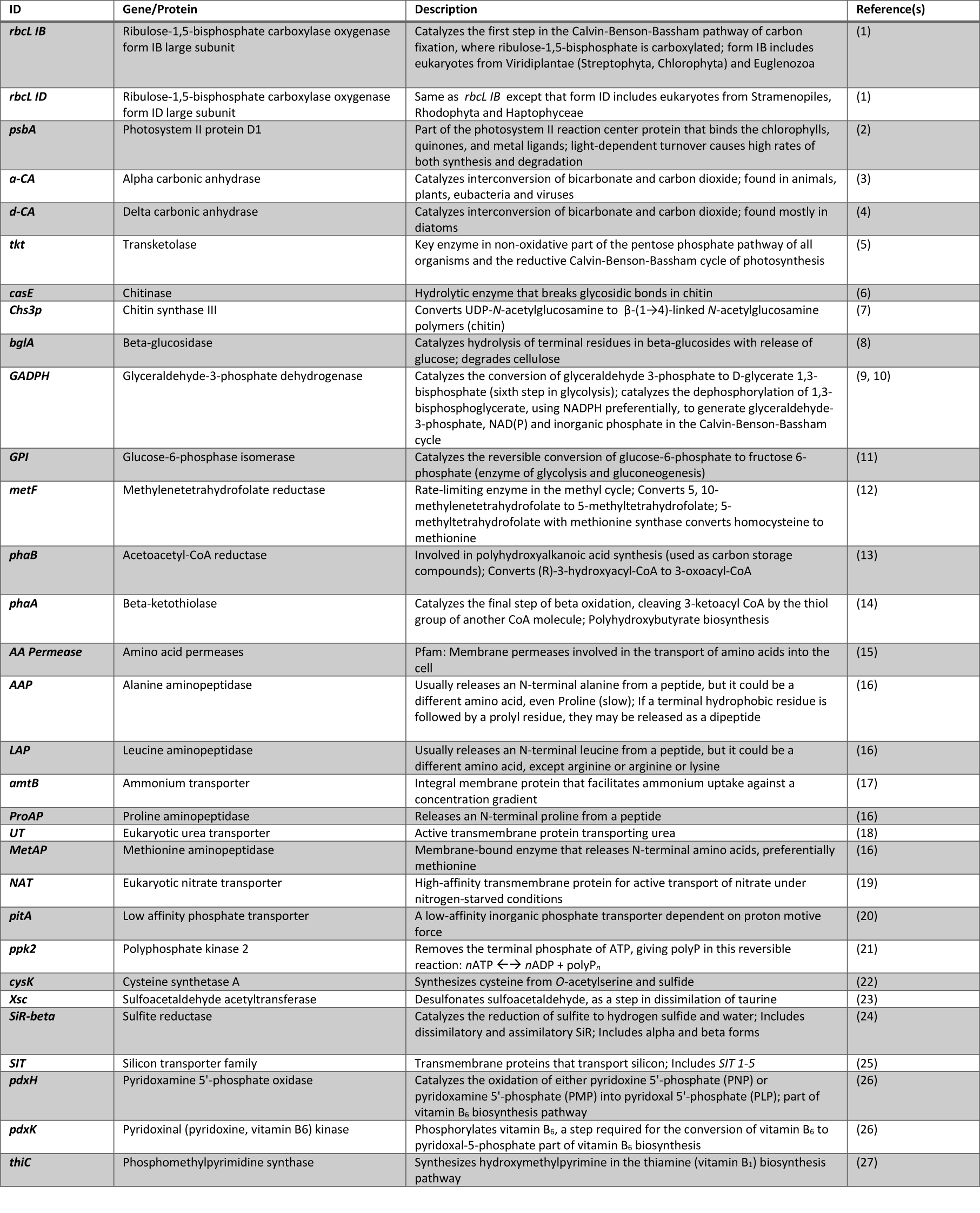

Supplement: S2 Table — (TIF) [file pone.0160929.s010.tif]
